# CT Perfusion Protocol for Acute Stroke Expedites Mechanical Thrombectomy

**DOI:** 10.7759/cureus.4546

**Published:** 2019-04-26

**Authors:** Matthew Jenson, Jeremiah Libby, Erik Soule, Sukhwinder J Sandhu, Peter J Fiester, Dinesh Rao

**Affiliations:** 1 Radiology, University of Florida College of Medicine, Jacksonville, USA; 2 Interventional Radiology, University of Florida College of Medicine, Jacksonville, USA; 3 Neuroradiology, Mayo Clinic, Jacksonville, USA; 4 Neuroradiology, University of Florida Health, Jacksonville, USA

**Keywords:** stroke, endovascular revascularization, magnetic resonance imaging, computed tomography perfusion, mechanical thrombectomy

## Abstract

The evaluation of a patient suspected of having an acute cerebrovascular accident is initiated with computed tomography (CT) and computed tomography angiogram (CTA) cross-sectional imaging of the head. Eligible patients may subsequently receive magnetic resonance imaging (MRI) utilizing a hyperacute stroke protocol. Clinical and imaging selection criteria are used to assess candidates for possible thrombectomy or thrombolysis. Prompt restoration of flow to ischemic regions of the cerebrum may result in improved neurological outcomes. Reducing delays in diagnosis and treatment remains paramount to effective treatment of ischemic cerebrovascular events. In an effort to expedite intra-arterial intervention, we replaced our institutional MRI protocol with a CT perfusion protocol. The amount of time the patient spent undergoing imaging was measured with each protocol and is referred to as "stroke imaging time." The purpose of this study was to compare the difference in the amount of time patients spent undergoing imaging when the acute stroke workup was performed with MRI vs. CT perfusion. Stroke imaging time decreased from an average of 158 minutes to 81 minutes (49%) by substituting CT perfusion for MRI. Utilizing CT perfusion in lieu of MRI in the hyperacute stroke protocol may expedite intra-arterial intervention.

## Introduction

The paradigm of acute cerebrovascular accident (CVA) holds that "time is tissue." This is widely accepted in acute ischemic disorders, including acute limb ischemia, acute myocardial infarction, and acute CVA, which are generally treated as endovascular surgical emergencies. Time to reperfusion has been shown to correlate with the probability of obtaining functional independence after endovascular therapy for CVA [1]. Recent studies have suggested that select patients can undergo mechanical thrombectomy beyond the traditional six-hour window [2]. The National Institute of Health (NIH) stroke scale is a clinical tool performed to assess the severity of acute stroke symptoms. Patients are awarded increasing points corresponding to their level of neurologic disability in 15 clinical domains at presentation. The NIH scale has shown a correlation with the infarct volume measured on computed tomography (CT) and magnetic resonance imaging (MRI), as well as a correlation with clinical outcomes. Patients with presenting scores of >14 were likely to require inpatient rehabilitation, and those with scores >15 at presentation were unlikely to achieve an excellent neurologic outcome at three months [3]. One strategy to improve outcomes in stroke care involves expediting the progression from presentation with stroke symptoms to time of groin puncture (GP) for endovascular thrombectomy.

Undergoing MRI was hypothesized to delay endovascular thrombectomy for eligible patients who presented with neurologic deficits related to acute and possibly developing CVAs. Due to prolonged scanning times, the need to lay still, screening checklists, and other impediments, delays occurred during the diagnostic workup of acute stroke patients. The hyperacute stroke imaging protocol was modified, replacing MRI with CT perfusion (CTP) in 2017. This was done to improve the diagnostic workup and expedite thrombectomy for patients with acute CVA. With the goal of measuring the effect of substituting CTP for MRI for evaluation of acute stroke patients, a new metric called "stroke imaging time" (SIT) was created. As the window to perform intra-arterial intervention after the onset of stroke symptoms was recently extended from six to 24 hours, a metric that controls for outside factors and compares only the time spent obtaining and evaluating imaging studies prior to intervention was needed. SIT begins when the CT technologist finalizes the initial non-contrast head CT and makes it available for viewing by the radiologist. When the radiologist notifies the ordering provider of the results of MRI or CTP, SIT is concluded. SIT can thus be used as a benchmark for time of radiologic assessment of the patient’s eligibility for intra-arterial therapy. SIT was found to be reduced by 49%, utilizing CTP instead of MRI in the hyperacute stroke imaging protocol.

## Materials and methods

All stroke patients who underwent mechanical thrombectomy from 2016-2018 at University of Florida Health Jacksonville were included for analysis. The primary time points evaluated include last time seen normal (LTSN), time of presentation with stroke symptoms (Door), and groin puncture time (GP). Stroke imaging time was created as a metric to evaluate the amount of time the patient spends undergoing imaging prior to intervention. SIT begins when the screening non-contrast head CT is available to the radiologist for viewing and ends when the radiologist provides the results of the follow-up confirmatory study to the ordering provider (MRI or CT perfusion). A comparison between stroke imaging time for patients who underwent MRI and patients who underwent CT perfusion was hypothesized to reveal a decrease in diagnostic time when CT perfusion was utilized.

During the time period corresponding to the MRI cohort, when MRI was utilized but not CTP, 16 patients underwent mechanical thrombectomy. Four of these patients did not obtain an MRI: two had MRI incompatible hardware, one patient was too large to fit in the MRI bore, and one patient did not undergo MRI for unknown reasons. These four patients who did not undergo MRI were excluded from the study. Additionally, two patients in the MRI cohort obtained their MRI after undergoing mechanical thrombectomy. As the focus of this study was the time it takes patients to navigate diagnostic imaging prior to intervention, these patients were also excluded from the study, leaving 10 patients in the MRI cohort. During the time period of the CTP cohort, when CTP was utilized but not MRI, 17 patients underwent mechanical thrombectomy. All of these patients underwent CTP but not MRI prior to mechanical thrombectomy and were included in the CTP cohort. For the 10 patients in the MRI cohort, result notification times were all documented and thus utilized in the analysis. In the 17 patients in the CTP cohort, the time the CTP results were notified to the ordering provider was not available in six patients. For these six patients, the time the preliminary report made by the radiology resident was made public was used as a proxy for the notification time.

## Results

**Figure 1:**
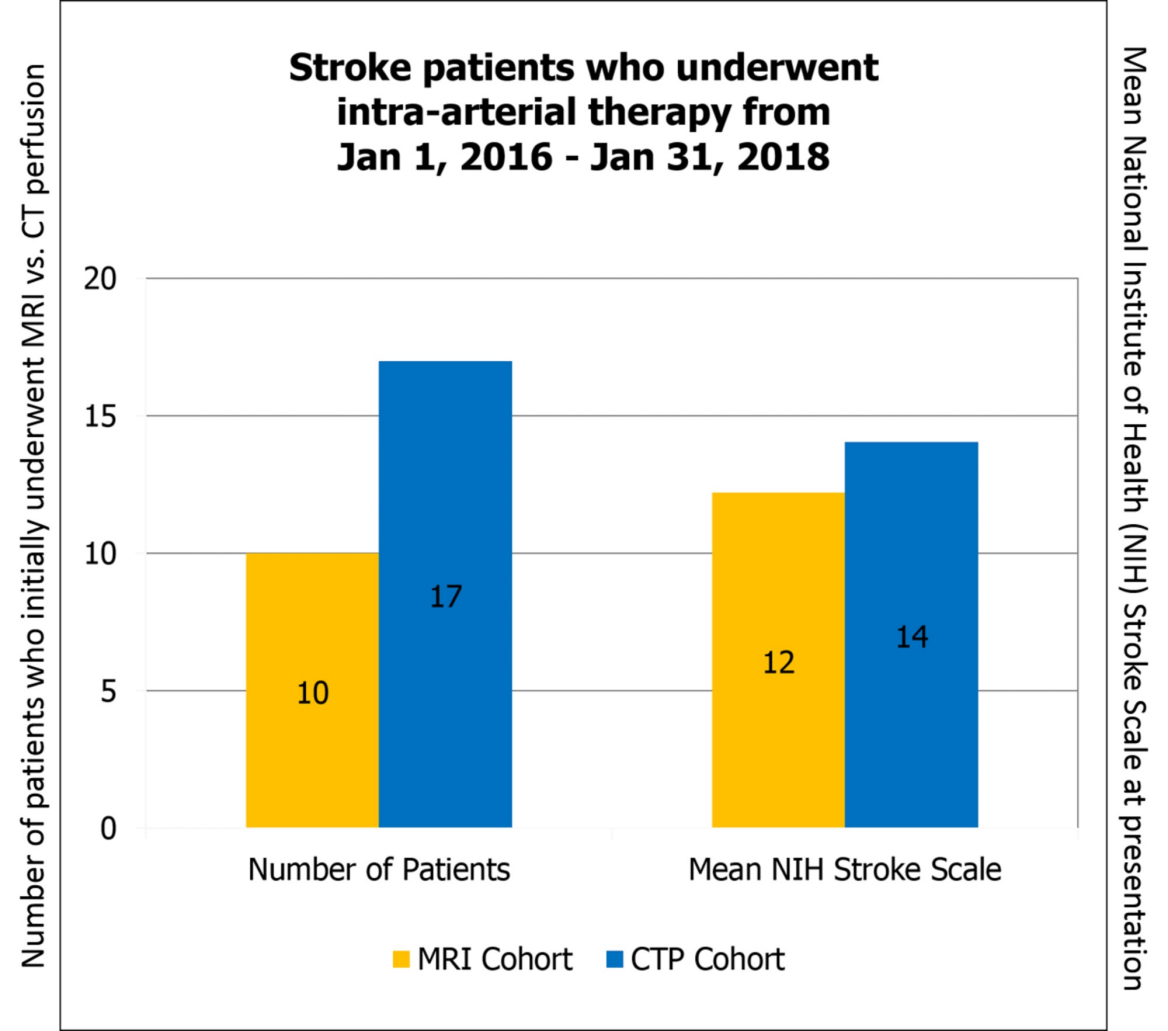
Patients who underwent mechanical thrombectomy from 2016–2018 were included in this study. The MRI cohort contains 10 patients and CTP cohort contains 17 patients. The mean NIH stroke scale was 12 in the MRI cohort and 14 in the CTP cohort. MRI: magnetic resonance imaging; CTP: computed tomography perfusion; NIH: National Institute of Health

The 10 patients in the MRI cohort had an average NIH score of 12. This compares with the 17 patients in the CTP cohort who had an average NIH score of 14 (Figure 1).

**Figure 2:**
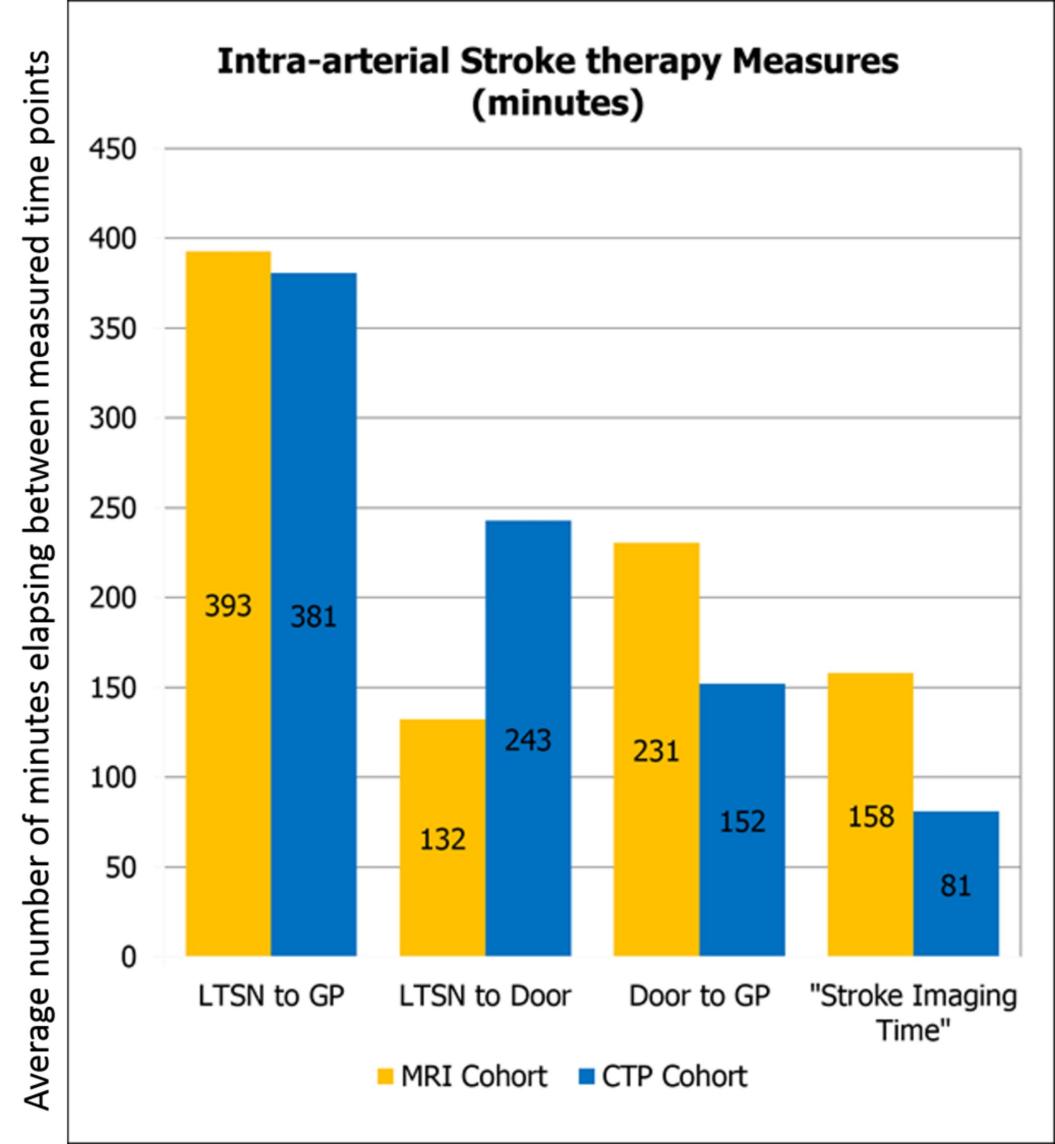
The time points utilized include LTSN, GP, and Door. Also included is the benchmark “stroke imaging time,” which includes the period of time between when the CT technologist finalizes the initial noncontrast CT head until when the ordering provider is notified of the results of the MRI or CTP. LTSN: last time seen normal; GP: groin puncture; Door: door time; CT: computed tomography; MRI: magnetic resonance imaging; CTP: CT perfusion

LTSN to GP time was 393 minutes on average in the MRI cohort, which decreased to 381 minutes in the CTP cohort. LTSN to door time was 132 minutes on average in the MRI cohort and was 243 minutes in the CTP cohort. The window to perform intra-arterial intervention increased from six hours after onset of symptoms to 24 hours after onset of symptoms while this study was taking place, thus the patients undergoing CT perfusion tended to present in a more delayed manner. Door to GP time changed from a mean of 231 minutes in the MRI cohort to 152 minutes in the CTP cohort. SIT decreased from 158 minutes in the MRI cohort to 81 minutes in the CTP cohort (Figure 2). 

## Discussion

CTP utilizes spiral CT scanning to track a bolus of iodinated contrast throughout the cerebral vasculature. Various parameters are measured and compared to ratios seen in normally perfused brain tissue. Alterations in cerebral blood flow (CBF), cerebral blood volume (CBV), time to peak (TTP), and mean transit time (MTT) may be altered in the setting of an acute CVA or hypoperfusion. A significantly decreased CBF and CBV combined with increased TTP and MTT compared to the normal contralateral side reflects alterations in physiology related to the "ischemic core" or irreversibly damaged tissue caused by CVA. Collateral vessels can provide some flow to ischemic tissue in the setting of primary arterial occlusion. If the CBF is significantly decreased and CBV is normal or increased, this may indicate hypoperfused brain tissue or the "ischemic penumbra." This area of cerebrum peripheral to the core infarcted area can be salvaged by timely intervention. TTP and MTT increases reflect the contrast taking collateral routes in an attempt to supply the ischemic tissue [4]. CT perfusion has been shown to provide clinicians with valuable information that may guide endovascular therapy and influence outcomes after intervention [5-7].

The interpretation of CTP may be confounded by the presence of vasospasm, seizure, vascular stenosis, microvascular ischemia, and small or chronic infarction [8]. CTP was shown to be less accurate than diffusion-weighted imaging (DWI) via MRI for estimating the volume of the "ischemic core" of the infarcted zone [9]. Thus, MRI remains the gold standard for determining which area of the cortex is irreversibly damaged. A large area of irreversible infarct and a small area of "ischemic penumbra" or peripheral zone of reversible ischemia is considered a contraindication for endovascular reperfusion due to the risk of hemorrhage and low likelihood of a good outcome. CTP, however, has been shown to be useful in clinical practice because it can elucidate the presence of a large penumbral zone, which is a predictor of good neurologic outcome after reperfusion [10].

Substituting CTP for MRI has resulted in decreased door to GP times for eligible stroke patients. Under the new protocol, the door to GP time was reduced by 79 minutes. SIT decreased by 77 minutes. These improvements can largely be explained by the shorter amount of time it takes patients to undergo imaging and for results to be communicated back to the ordering provider. It has been shown that time to groin puncture can be reduced by performing CTP in the community before transfer to a stroke center for treatment [11]. LTSN to GP times remained relatively unchanged, which is likely related to the recent widening of the stroke window timeframe for intra-arterial intervention. Increased LTSN to door times provides further evidence of this. While more patients beyond the traditional six-hour window are being considered for intra-arterial therapy, the time it takes to image patients and determine their eligibility for intra-arterial intervention has been improved using CTP in lieu of MRI.

In practice, using MRI to triage stroke patients for endovascular thrombectomy is fraught with many obstacles that cause SIT to be delayed. The MRI takes considerably more time to obtain as compared to a CTP examination, which only takes about two minutes to complete and can potentially be done while the patient is already in the CT scanner for prerequisite non-contrast head CT and CTA. In addition to moving the patient to a different machine to undergo MRI, there are checklists that must be completed by the MRI technologist to ensure that MRI is not contraindicated in the patient due to incompatible devices and so on. Completing these checklists and lying still for an MRI may be difficult for a patient with multiple neurologic defects demonstrated by an average NIH stroke scale of 12-14 in this study. It is reasonable to conclude from these data that switching from MRI to CTP in the acute stroke imaging protocol has expedited the diagnostic workup and reduced the door to groin puncture times for patients with acute CVA. As CTP can be used to obtain adequate information to recommend and proceed to stroke intervention, it may be the preferred imaging modality in acute CVA workups.

## Conclusions

Utilizing CTP in the acute stroke imaging protocol has led to improved intra-arterial intervention times for eligible CVA patients. Under the new protocol, door to GP time was reduced by 79 minutes. SIT, the benchmark created to measure the time spent in imaging prior to intervention, decreased by 77 minutes. Thus, the improvement in door to GP time can largely be explained by the shorter amount of time it takes patients to undergo the imaging protocol and receive imaging results by replacing MRI with CTP. LTSN to GP times remained relatively unchanged, which is likely related to the recent widening of the stroke window timeframe for intra-arterial intervention. Increased LTSN to door times provide further evidence of this. Although clinicians are becoming more aggressive in selecting acute CVA patients who are eligible for endovascular intervention, the time to initiate intra-arterial therapy from presentation was improved using CTP instead of MRI.
